# Suicide in India: The need for a national policy

**DOI:** 10.4103/0019-5545.31626

**Published:** 2006

**Authors:** S.D. Manoranjitham, R. Jayakaran, K.S. Jacob

**Affiliations:** * Professor of Psychiatric Nursing, Department of Psychiatry, Christian Medical College, Vellore 63202, Tamil Nadu; ** Professor of Community Nursing, Department of Psychiatry, Christian Medical College, Vellore 63202, Tamil Nadu; *** Professor of Psychiatry, Department of Psychiatry, Christian Medical College, Vellore 63202, Tamil Nadu

Sir,

This refers to the article on psychological autopsy of suicide by Khan *et* al.^1^ The documented suicide rates in India vary from 6.8 to 58.3 per 100,000.^2^ These studies have used data collected from police records, which tend to under-report suicides. The families of suicide victims usually do not want post-mortems to be done because of the fear of mutilation of the body, the time-consuming nature of the process and the stigma involved. Death by suicide is often reported as due to illness or accident to avoid police investigation.

Recent reports from Vellore suggest that the suicide rate in India is grossly under-reported. These studies employed verbal autopsies to diagnose suicide and employed a population-based community health programme to calculate rates. The average annual suicide rate for the general population was about 95 per 100,000;^3^,^4^ 148/100,000 and 58/100,000 for young women and men, respectively;^5^ and 189/ 100,000 in the elderly.^6^ Studies from Vellore also report that major risk factors for suicide include presence of chronic stress and precipitating life events rather than severe mental disorders.^4^ The high rates of suicides tend to confirm the impression of many researchers that the suicide rate in India is much higher than that reported by national suicide statistics. The authors argue that the high rates are not peculiar to the study population and they reflect more accurate data collection. It is possible that similar situations exist in other parts of India as well.

Reportedly high rates of suicides suggest the need for a national strategy which will raise awareness and help make suicide prevention a national priority.^7^ A national strategy to prevent suicide will need to have a comprehensive approach that encompasses the promotion, coordination and support of activities which will be implemented across the country at national, regional, and community levels. Developing a national strategy provides an opportunity to bring together many sectors of society—government, public health, education, religion, non-governmental organizations, users and advocacy groups. Programmes for suicide prevention for the Indian context will have to be designed, implemented and evaluated on a mass scale. Such effort at implementing and evaluation will need to be coordinated at the regional, state and district levels. The coordination between academic and clinical professionals, and partnership between public and private enterprises, government and non-governmental organizations will be cardinal to the success in suicide prevention. Organizations working in health, education, social welfare, police, and the judiciary will have to be involved in the planning and organization of such policy and services. Changes in the law would require legislative and judicial support. The programmes will need to develop interdisciplinary support to evolve a broad-based approach for suicide prevention and research.
